# Publisher Correction: Cryo-EM structure of the sodium-driven chloride/bicarbonate exchanger NDCBE

**DOI:** 10.1038/s41467-021-26412-7

**Published:** 2021-10-13

**Authors:** Weiguang Wang, Kirill Tsirulnikov, Hristina R. Zhekova, Gülru Kayık, Hanif Muhammad Khan, Rustam Azimov, Natalia Abuladze, Liyo Kao, Debbie Newman, Sergei Yu. Noskov, Z. Hong Zhou, Alexander Pushkin, Ira Kurtz

**Affiliations:** 1 Department of Medicine, Division of Nephrology, David Geffen School of Medicine, University of California, Los Angeles, CA USA; 2 Electron Imaging Center for Nanomachines, California NanoSystems Institute, University of California, Los Angeles, CA USA; 3 Centre for Molecular Simulation, Department of Biological Sciences, University of Calgary, Calgary, Canada; 4 Department of Microbiology, Immunology and Molecular Genetics, University of California, Los Angeles, CA USA; 5 Brain Research Institute, University of California, Los Angeles, CA USA

**Keywords:** Sodium, Membrane proteins, Computational biophysics, Cryoelectron microscopy

Correction to: *Nature Communications* 10.1038/s41467-021-25998-2, published online 28 September 2021.

The original version of this Article contained an error in the ‘Methods’ section, which incorrectly read ‘<Na^+^‒anctional anal3‾anctional analysist assays>’. The correct version states ‘<Na^+^‒driven Cl‾/HCO3‾ exchange transport assays>’ in place of ‘<Na^+^‒anctional anal3‾anctional analysist assays >’. This has been corrected in both the PDF and HTML versions of the Article.

