# Supplementary material for: Cryo-EM structure of the sodium-driven chloride/bicarbonate exchanger NDCBE
Source: Nat Commun. 2021 Sep 28;12:5690. doi: 10.1038/s41467-021-25998-2 (PMC8478935; doi:10.1038/s41467-021-25998-2)
Supplement: Supplementary file 1 — Supplementary Information [file 41467_2021_25998_MOESM1_ESM.pdf]

Supplementary information

Cryo-EM Structure of the Sodium-driven Chloride/Bicarbonate Exchanger NDCBE

Wang et al.

Supplementary Figures

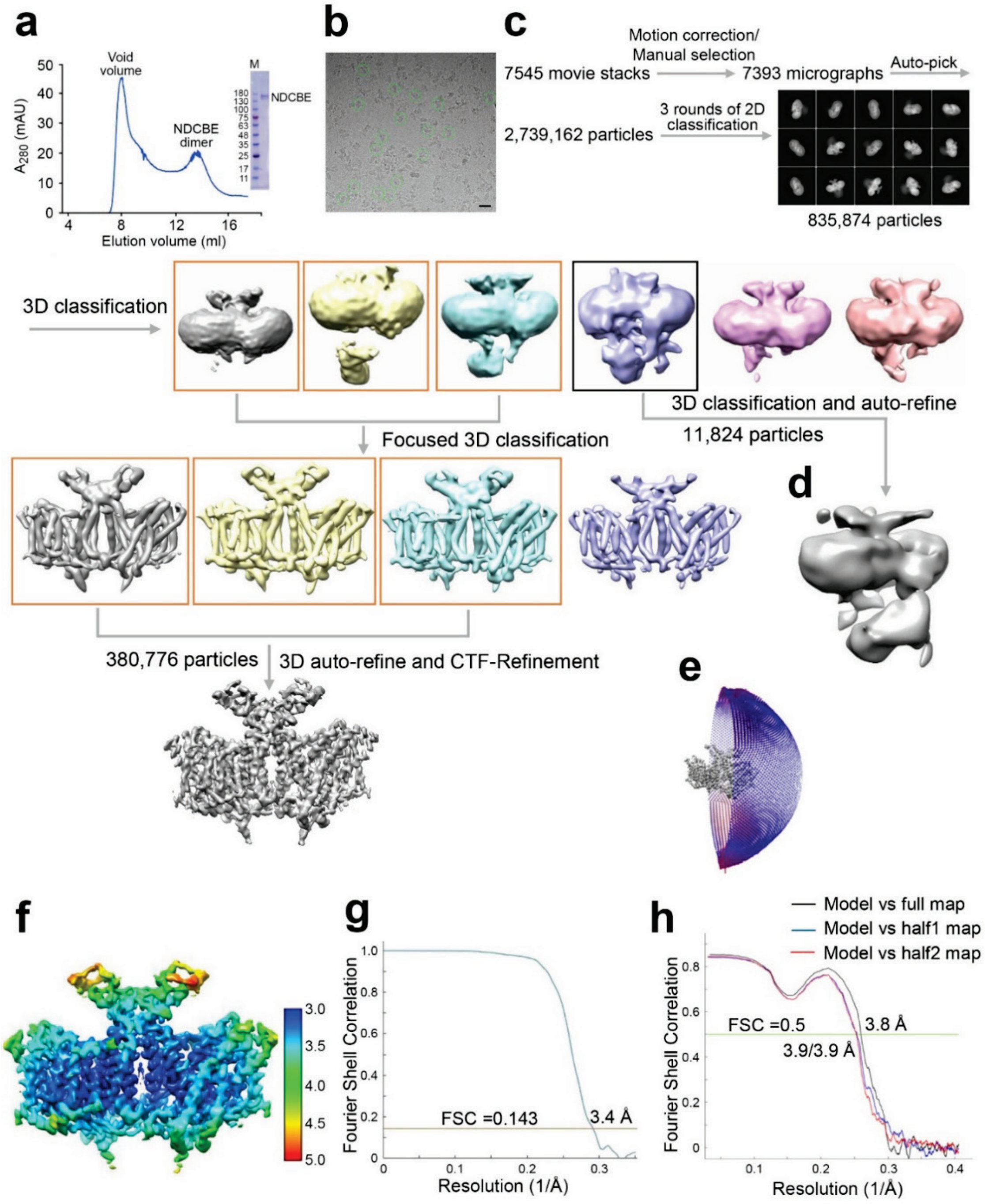

Supplementary Figure 1. Structure determination of NDCBE. a Gel-filtration of NDCBE in a buffer containing Na^+, Cl^-, HCO_3^-, Tris-HCl, with LMNG (pH 7.4) and Coomassie staining of purified NDCBE (n = 4 biologically independent experiments). Source data are provided as a Source Data file. **b** Representative cryo-EM micrograph of NDCBE in LMNG (n = 7,545 micrographs; scale bar = 20 nm). **c** Flowchart of image processing for NDCBE. **d** Density map of full-length NDCBE structure. **e** Angular distribution map of NDCBE. **f** Local resolution analysis of the NDCBE cryo-EM map using Resmap. **g** The Gold standard FSC curve of the final 3D reconstruction. **h** The FSC curves for cross-validation between the maps and the model.

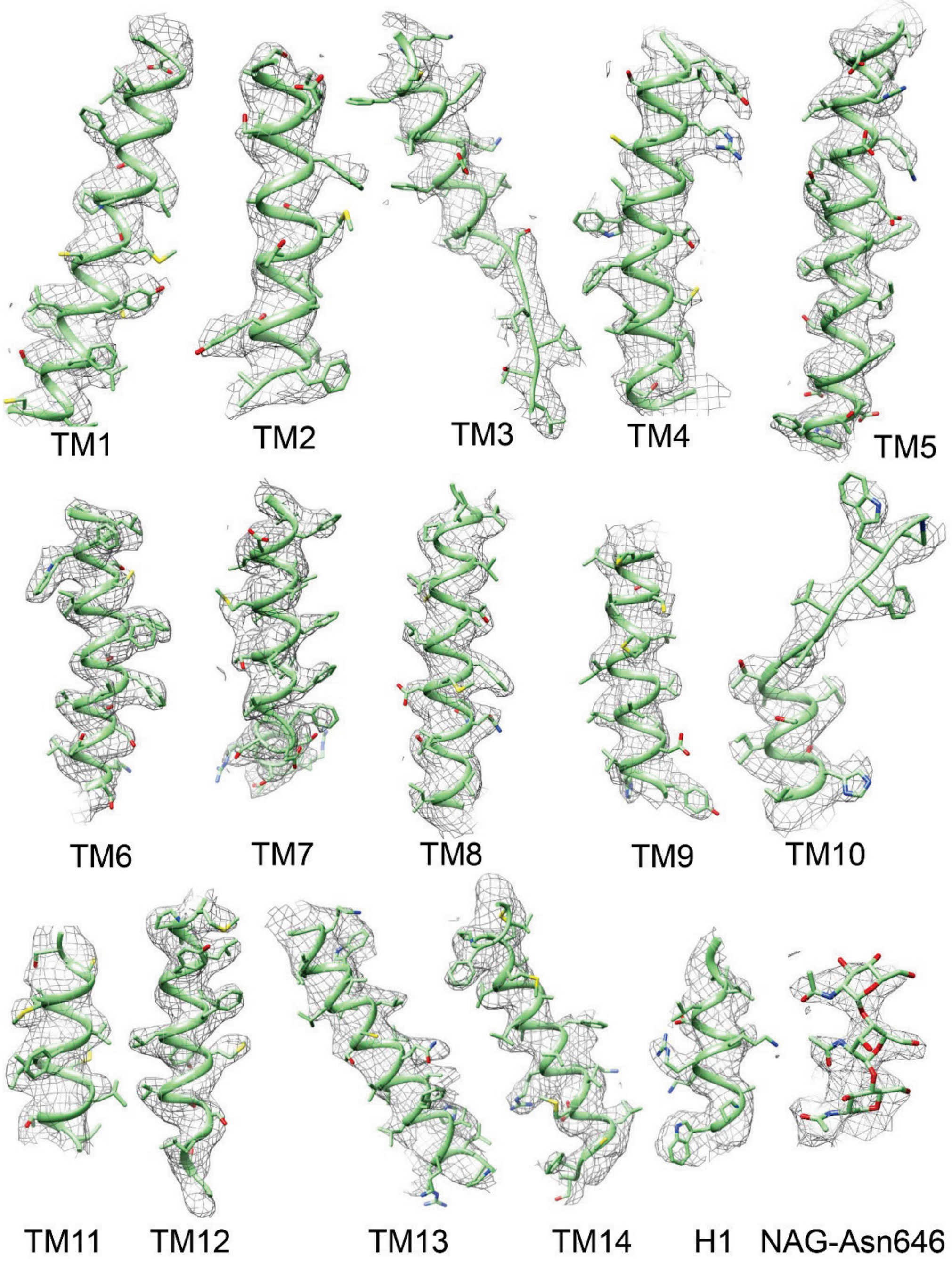

Supplementary Figure 2. Cryo-EM density maps of NDCBE TMD regions superposed with atomic models. Representative maps at 14 TMs of NDCBE, H1 and NAG-Asn646 are shown.

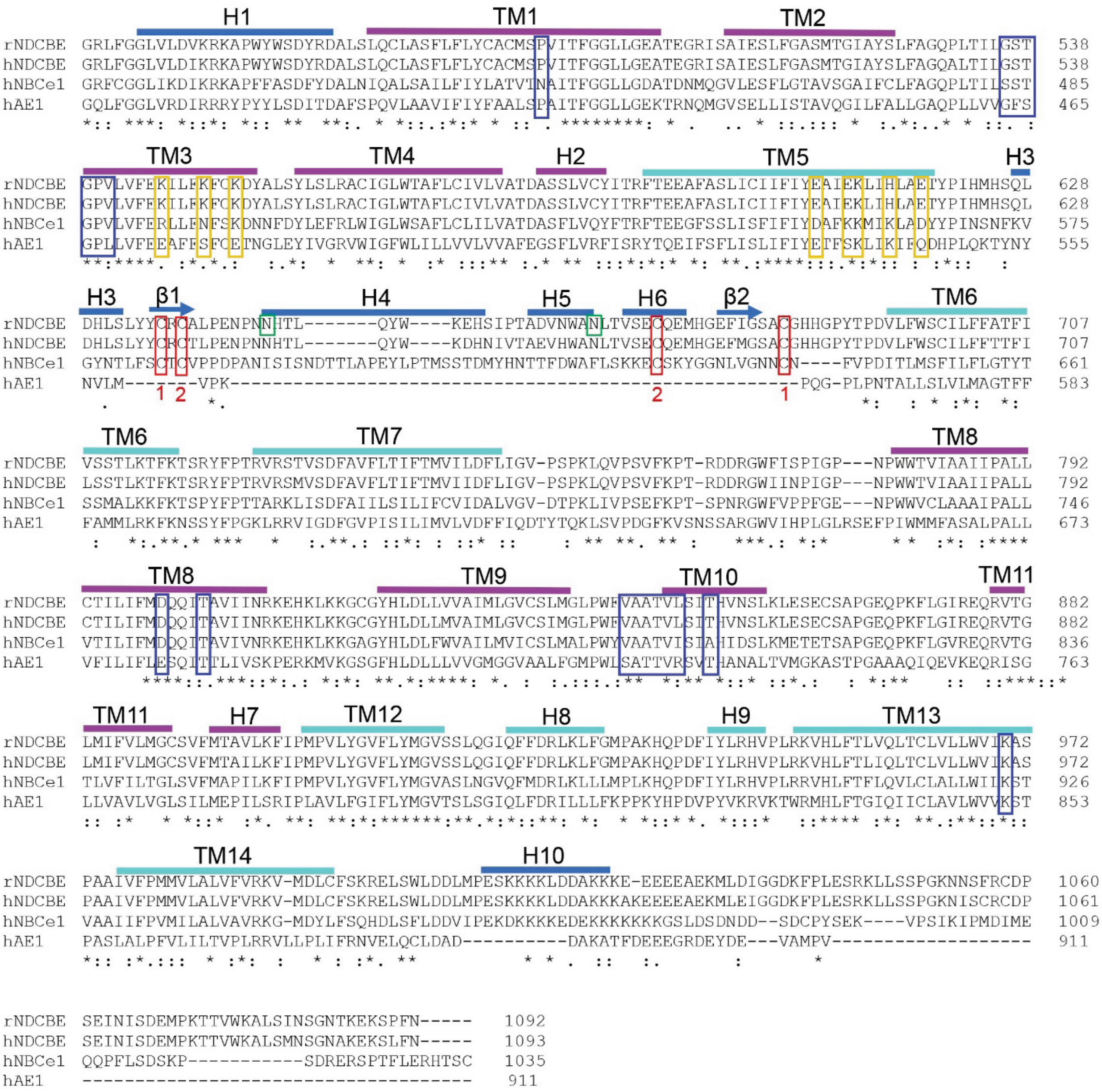

Supplementary Figure 3. Sequence alignment of NDCBE, AE1 and NBCe1. The Clustal Omega program[62] was used for the alignment. Secondary structure assignments are based on the cryo-EM structure of NDCBE. The sequence alignment of the N-terminal domain is not shown. Red boxes and numbers mark the cysteine residues that form disulfide bonds and green boxes show the glycosylation sites of the asparagine residues in EL3. Blue boxes highlight the residues from the binding pockets of AE1, NBCe1 and NDCBE (presented in Figs. 2,3,5). Brown boxes show the most pronounced differences in the charged residues lining TMs 3 and 5 of the ion permeation pathways (presented in Fig. 3a).

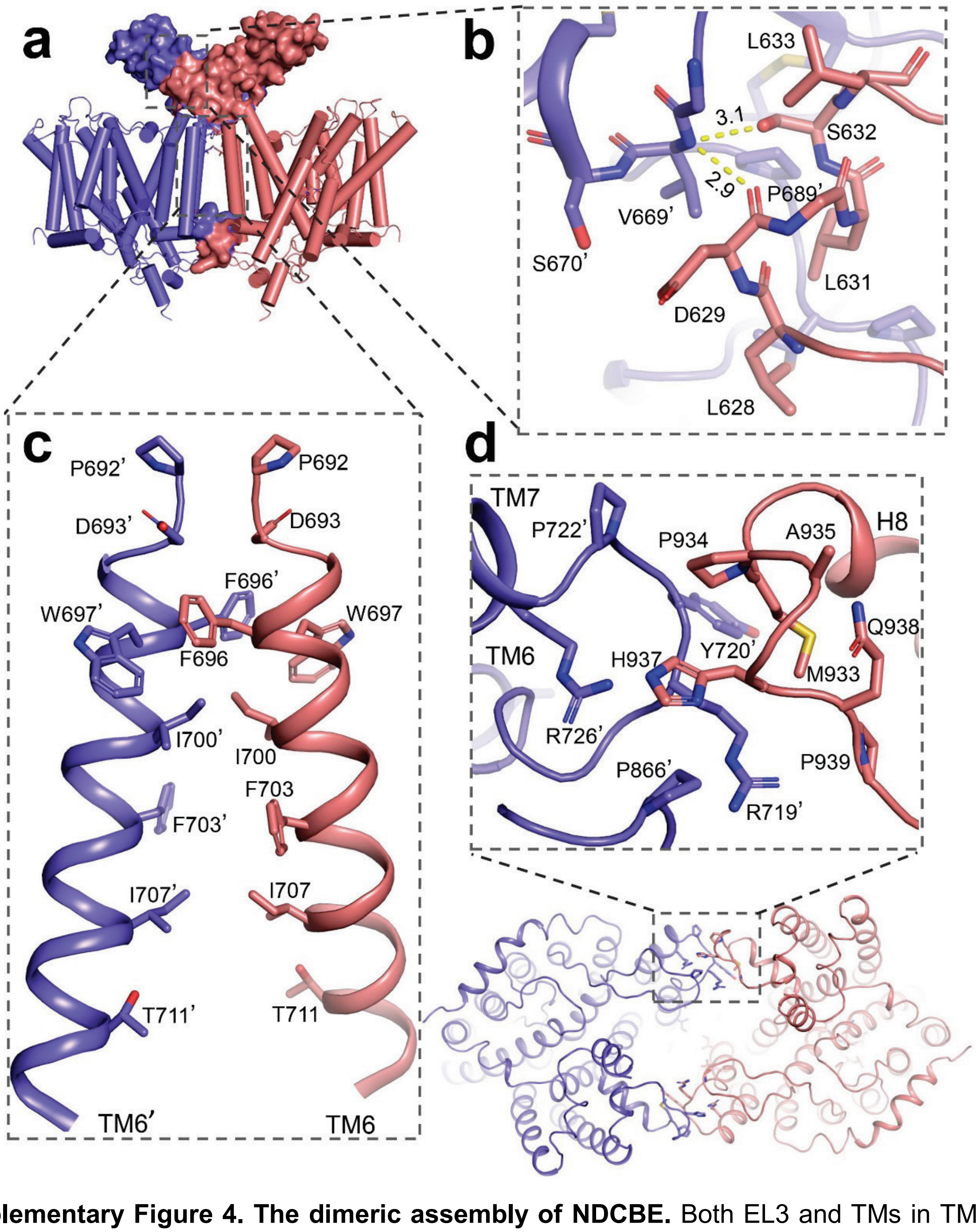

Supplementary Figure 4. The dimeric assembly of NDCBE. Both EL3 and TMs in TMD are involved in dimerization.

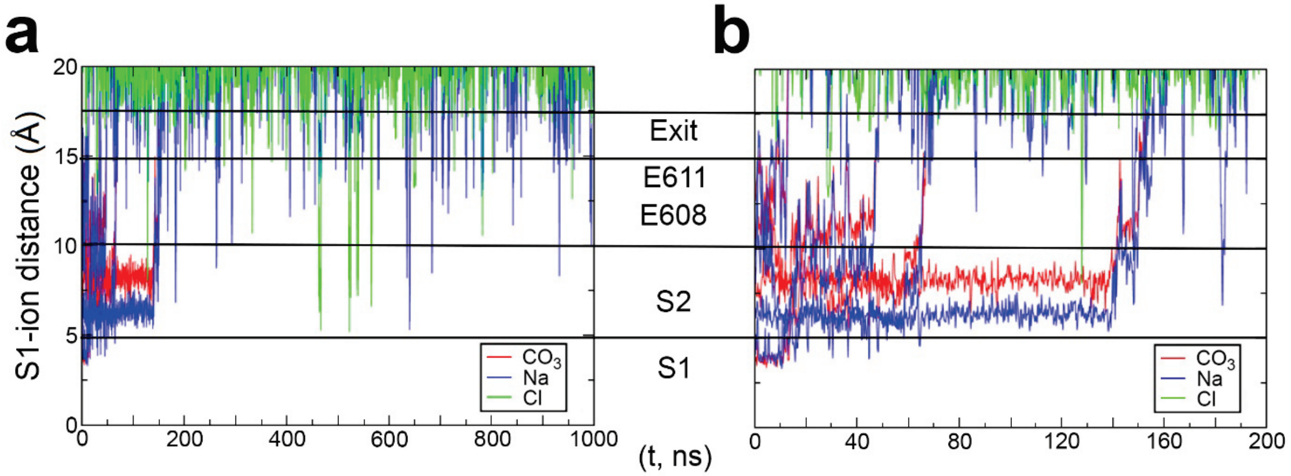

Supplementary Figure 5. Ion time series for the 1μs MD trajectories. a Aggregated ion time series for the four 1 μs MD trajectories (Trials 1-4 in Table S1). The time series for CO_3^{2-}, Na^+, and Cl^- are shown in red, blue, and green color respectively. The S1-ion distance is evaluated as the minimum distance between the center of an ion of a certain type (Na^+, Cl^-, HCO_3^-, CO_3^{2-}) and the C_α atom of residue Ala846 from site S1. **b** Same as (**a**), however only the first 200 ns of the trajectories are shown. The relative position of sites S1 and S2, as well as residues of importance (Glu608, Glu611) and the cavity exit with respect to the C_α atom of residue Ala846 from site S1 are also indicated in the figure. All trajectories start from pre-equilibrated MD simulations with a Na^+-CO_3^{2-} pair in the S1/S2 binding pocket. The initial position of the Na^+-CO_3^{2-} differ in each trajectory. The CO_3^{2-} and Na^+ from the ion pair eventually dissociate together from the binding pocket and exit the OF cavity. Afterwards Na^+ and Cl^- ions from the solution occasionally permeate the cavity and reside briefly in the areas of site S2 (Cl^-) or in the vicinity of two acidic residues on TM5, Glu608 and Glu611 (Na^+).

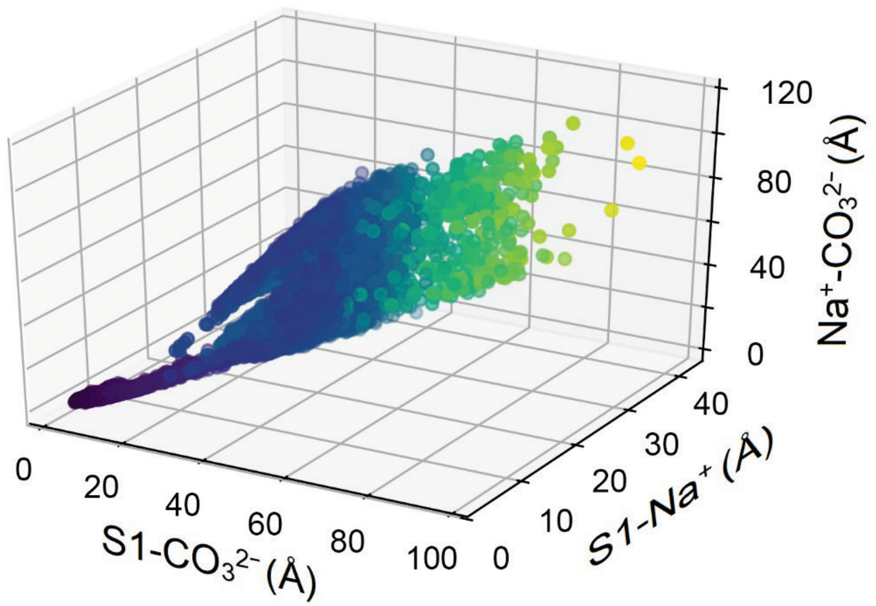

Supplementary Figure 6. Correlation of initially bound Na^+ and CO_3^{2-}. Aggregated ion correlation graph for the four 1 μs MD trajectories (Trials 1-4 in Table S1). S1-Na^+ and S1-CO_3^{2-} are the distances of the initially bound Na^+ and CO_3^{2-} ions to the C_α atom of residue Ala846 from site S1, respectively. Na^+-CO_3^{2-} is the distance between the initially bound Na^+ and CO_3^{2-} ions. As the linear dependence and the small Na^+-CO_3^{2-} distance in the OF cavity (the area enclosed by 20 Å S1-Na^+ x 20 Å S1-CO_3^{2-}) suggest, the two ions from the initially bound Na^+-CO_3^{2-} dissociate together from the binding pocket.

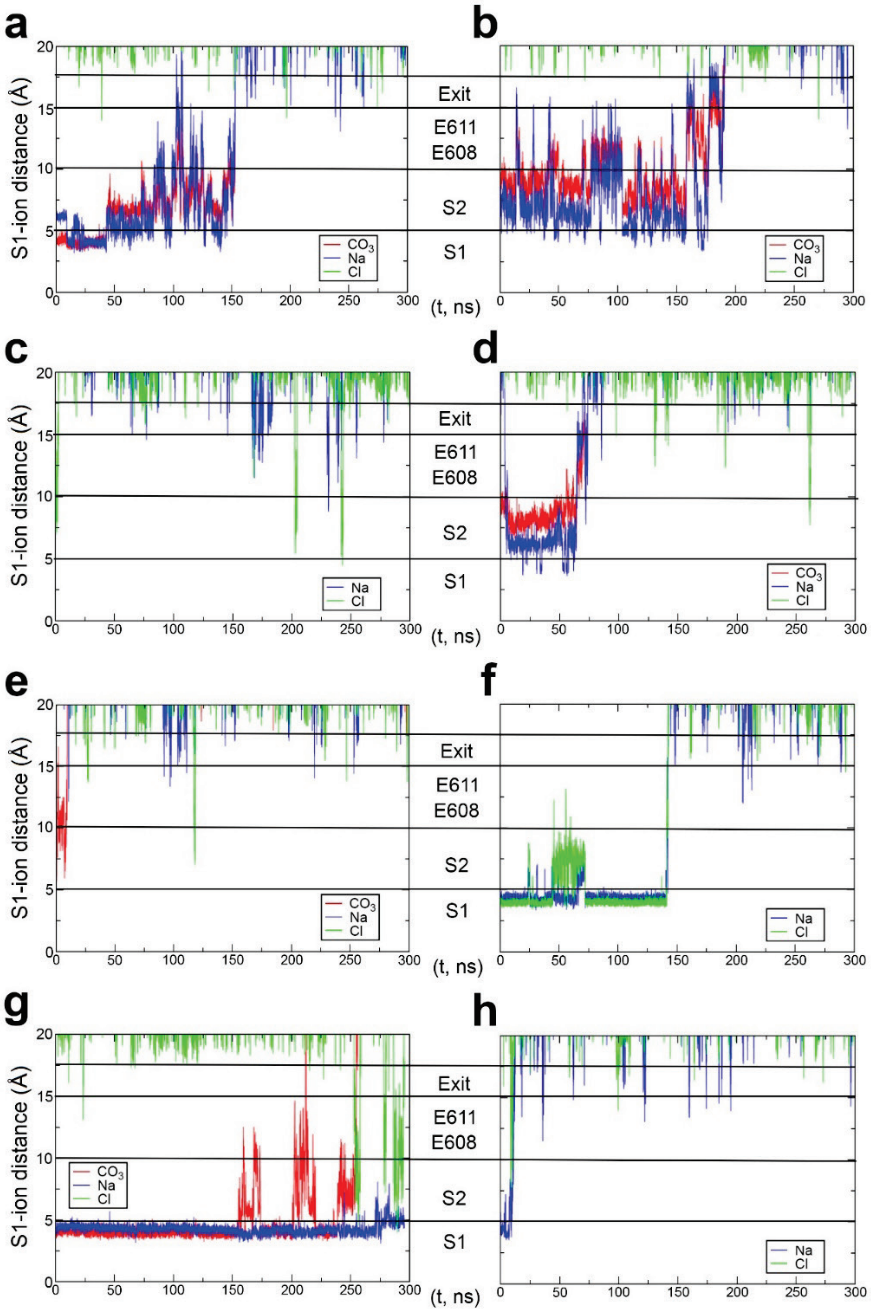

Supplementary Figure 7. Ion time series for the 300 ns MD trajectories with different ion loads presented in Table S1. **a** Trial 5 and **b** Trial 6 from Table S1 for NDCBE loaded with a Na^+-CO_3^{2-} pair in the area of site S1. **c** NDCBE loaded with a single Cl^- ion. **d** NDCBE loaded with a single CO_3^{2-} ion. **e** NDCBE loaded with a single HCO_3^- ion. **f** NDCBE loaded with a Na^+-Cl^- pair. **g** NDCBE loaded with a Na^+-HCO_3^- pair. **h** NDCBE loaded with a single Na^+ ion. The time series for CO_3^{2-} and HCO_3^- are shown in red while the time series for Na^+, and Cl^- are shown in blue and green, respectively. The S1-ion distance is evaluated as the minimum distance between the center of an ion of a certain type (Na^+, Cl^-, HCO_3^-, CO_3^{2-}) and the C_α atom of residue Ala846 from site S1. The relative position of sites S1 and S2, as well as residues of importance (Glu608, Glu611) and the cavity exit with respect to the C_α atom of residue A846 from site S1 are also indicated in the figure.

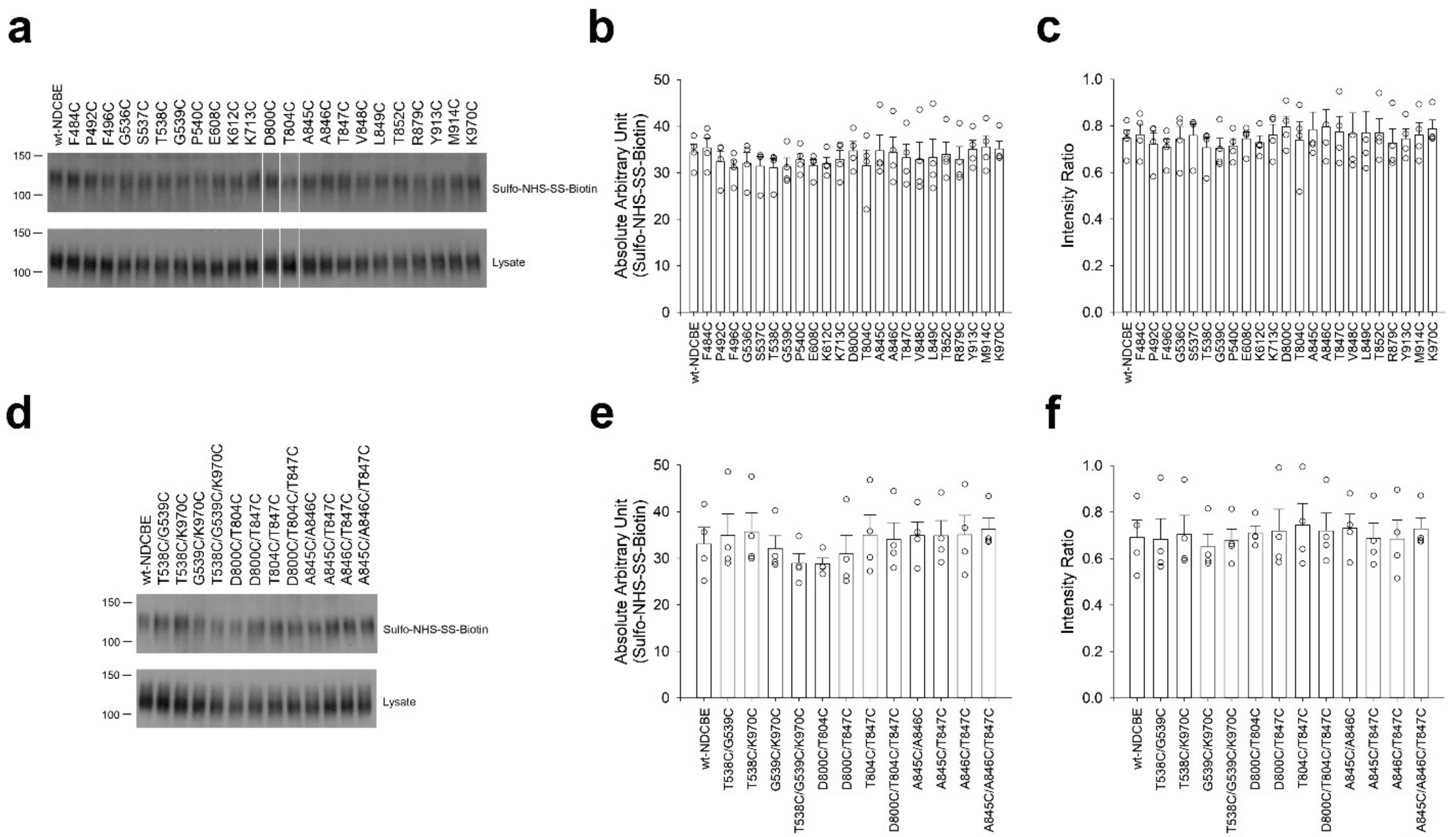

Supplementary Figure 8. Membrane expression of NDCBE mutants. a,d Representative experiments showing immunoblot analysis of cell-surface and cell-lysate expression of wt and mutant NDCBE proteins (n = 4 biologically independent experiments). The positions of molecular weight size markers (kDa) are shown on the left. Blot splicing is indicated with a vertical white line. Source data are provided as a Source Data file. **b,c,e,f** Densitometry analysis of cell-surface expression and the ratio of cell-surface to cell-lysate intensity of NDCBE protein expression (n = 4 biologically independent experiments). One-way ANOVA and Dunnett’s test were used to compare multiple study group means with wt-NDCBE. Mutant NDCBE data was not statistically different from wt-NDCBE. Results are depicted as mean ± SEM. Open circles represent individual data points. Source data are provided as a Source Data file.

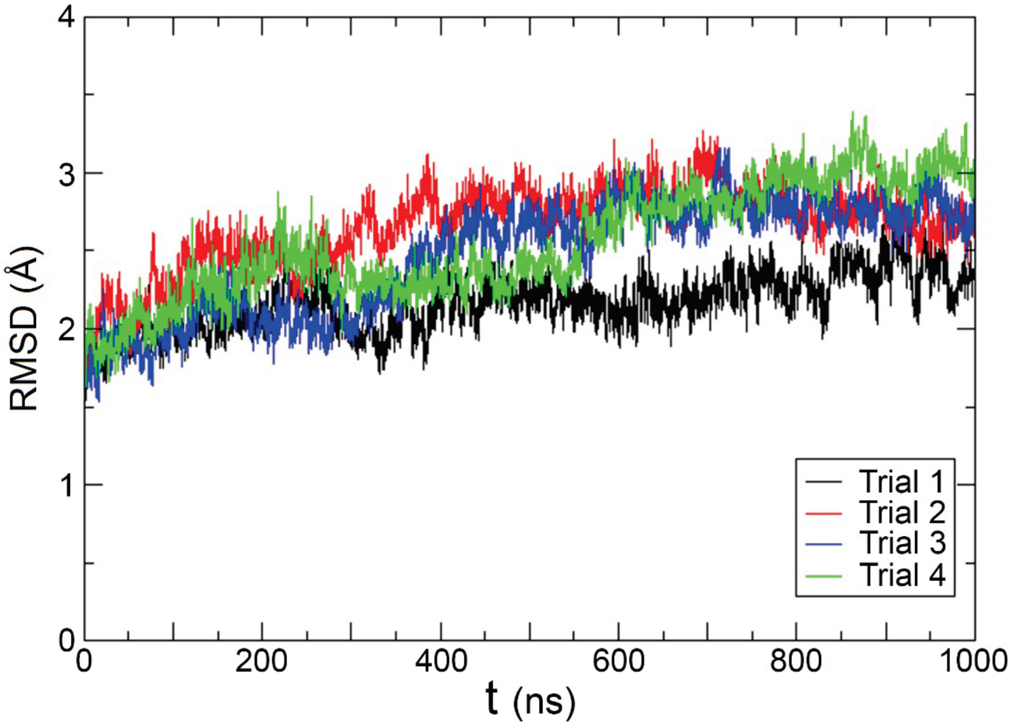

Supplementary Figure 9. Cα RMSD values calculated for the TMD portion of NDCBE obtained from the four 1 μs MD simulations of NDCBE. (Trials 1-4 in Table S1).

Supplementary Tables

Supplementary Table 1. Ion residence times (in ns) in the area of sites S1/S2 of NDCBE with different ion loads obtained from 1 μs and 300 ns MD simulations. The ion time series for the trajectories listed in Table S1 are presented in Supplementary Figures 5 and 7.

Ion load	MD length	Anion residence time, ns	Na^{+} residence time, ns
Na^{+} + CO_3^{2-} (trial 1)	1 μs	48.5	48.5
Na^{+} + CO_3^{2-} (trial 2)	1 μs	67	66
Na^{+} + CO_3^{2-} (trial 3)	1 μs	13	13
Na^{+} + CO_3^{2-} (trial 4)	1 μs	152.5	152.5
Na^{+} + CO_3^{2-} (trial 5)	300 ns	153	153
Na^{+} + CO_3^{2-} (trial 6)	300 ns	191	191
Cl^{-}	300 ns	3	-
CO_3^{2-}	300 ns	74*	-
HCO_3^{-}	300 ns	9	-
Na^{+} + Cl^{-}	300 ns	142	142
Na^{+} + HCO_3^{-}	300 ns	256	296**
Na^{+}	300 ns	-	12

*A portion of this simulation is presented in Supplementary Movie 2. The prolonged anion residence time here is the result of Na^{+} from the solution binding to Asp800 (see Supplementary Figure 7d) and stabilizing the CO_3^{2-} ion in analogy to the other Na^{+}- CO_3^{2-} simulations (Trials 1-6).

** Upon HCO_3^{-} departure from the binding pocket, Cl^{-} ions from the solution permeate to area of binding sites S1/S2, attracted by the Na^{+} ion which remains there (see Supplementary Figure 7g).

Supplementary Table 2. Free energy values (in kcal/mol) evaluated for different ion loads in site S1 of NDCBE. G_{hydr} is the absolute free energy of hydration for the individual anions. G_{site} is the absolute free energy of the interaction between the anion and the protein matrix (the Na^+ in the Na^+ bound systems is included in the protein matrix). ΔG_{bind} is the difference between the free energies of hydration and the free energies of interaction with the protein matrix. More negative ΔG_{bind} implies stronger ion binding to site S1 of NDCBE.

	Na^+- Cl^-	Na^+- HCO_3^-	Na^+-CO_3^{2-}	Cl^-	HCO_3^-	CO_3^{2-}
G_{hydr}	-	-	-	-81.3	-86.5	-272.5
G_{site}	-83.1	-89.8	-291.3	-75.1	-86.1	-282.4
ΔG_{bind}	-1.8	-3.3	-19.8	6.2	-0.4	-9.9
