# Supplementary material for: Cryo-EM structure of the sodium-driven chloride/bicarbonate exchanger NDCBE
Source: Nat Commun. 2021 Sep 28;12:5690. doi: 10.1038/s41467-021-25998-2 (PMC8478935; doi:10.1038/s41467-021-25998-2)
Supplement: Supplementary file 3 — Description of Additional Supplementary Files [file 41467_2021_25998_MOESM3_ESM.docx]

**File Name: Supplementary Data 1**

**Description:** PDB files representing the initial and final steps from the MD trajectories.

**File Name: Supplementary Movie 1**

**Description:** Dissociation of the Na^+^-CO_3_^2^¯ pair from the binding pocket of NDCBE. A Na^+^-CO_3_^2^¯ pair leaves the binding pocket of NDCBE (movie extracted from the 300 ns MD trajectory of Trial 5, Table S1). The residues in the binding pocket are shown as red (acidic residues), blue (basic residues), and cyan (polar and non-polar residues from sites S1 and S2, Fig.3) sticks. Hydrogen atoms are omitted for more clarity. The three charged residues (E608, D800, and K970) in the binding pocket are labeled for convenience. The ions are presented as purple (Na^+^), green (CO_3_^2^¯), and cyan (Cl¯) spheres. The protein matrix is shown in helical representation (white helices). During the dissociation process, Na^+^ loses contact with D800 and briefly interacts with E608 before both ions dissociate from site S2 and leave the binding pocket together.

**File Name: Supplementary Movie 2**

**Description:** Capture of a Na^+^ ion by a CO_3_^2^¯ ion bound in site S2. The CO_3_^2^¯ ion bound in site S2 spontaneously captures a Na^+^ from the surrounding solution and aids in Na^+^ binding to D800 and formation of a bound Na^+^- CO_3_^2^¯ pair in site S2 (movie extracted from the 300 ns MD trajectory of NDCBE loaded with a CO_3_^2^¯ ion, Table S1). The residues in the binding pocket are shown as red (D800), blue (K970), and white (polar and non-polar residues from sites S1 and S2, Fig.3) sticks. Hydrogen atoms are omitted for more clarity. The two charged residues (D800 and K970) in the binding pocket are labeled for convenience. The ions are presented as purple (Na^+^), green (CO_3_^2^¯), and cyan (Cl¯) spheres. The protein matrix is shown in helical representation (white helices).

**File Name: Supplementary Movie 3**

**Description:** Migration of Na^+^-CO_3_^2^¯ pair from site S2 to site S1. The Na^+^-CO_3_^2^¯ pair migrates spontaneously from site S2 to site S1 (movie extracted from the 300 ns MD trajectory of Trial 6, Table S1). The residues in the binding pocket are shown as red (D800), blue (K970), and cyan (polar and non-polar residues from sites S1 and S2, Fig.3) sticks and are labeled for convenience. Hydrogen atoms are omitted for more clarity. The ions are presented as purple (Na^+^) and green (CO_3_^2^¯) spheres. The protein matrix is shown in helical representation (transparent helices).
