# Supplementary material for: Cryo-EM structure of the sodium-driven chloride/bicarbonate exchanger NDCBE
Source: Nat Commun. 2021 Sep 28;12:5690. doi: 10.1038/s41467-021-25998-2 (PMC8478935; doi:10.1038/s41467-021-25998-2)
Supplement: Supplementary file 8 — Reporting Summary [file 41467_2021_25998_MOESM8_ESM.pdf]

nature research

Corresponding author(s): Ira Kurtz

Last updated by author(s): Sep 5, 2021

Reporting Summary

Nature Research wishes to improve the reproducibility of the work that we publish. This form provides structure for consistency and transparency in reporting. For further information on Nature Research policies, see our Editorial Policies and the Editorial Policy Checklist.

Statistics

For all statistical analyses, confirm that the following items are present in the figure legend, table legend, main text, or Methods section.

n/a	Confirmed	
☐	☒	The exact sample size (*n*) for each experimental group/condition, given as a discrete number and unit of measurement
☒	☐	A statement on whether measurements were taken from distinct samples or whether the same sample was measured repeatedly
☐	☒	The statistical test(s) used AND whether they are one- or two-sided<br>*Only common tests should be described solely by name; describe more complex techniques in the Methods section.*
☒	☐	A description of all covariates tested
☒	☐	A description of any assumptions or corrections, such as tests of normality and adjustment for multiple comparisons
☐	☒	A full description of the statistical parameters including central tendency (e.g. means) or other basic estimates (e.g. regression coefficient) AND variation (e.g. standard deviation) or associated estimates of uncertainty (e.g. confidence intervals)
☐	☒	For null hypothesis testing, the test statistic (e.g. *F*, *t*, *r*) with confidence intervals, effect sizes, degrees of freedom and *P* value noted<br>*Give P values as exact values whenever suitable.*
☒	☐	For Bayesian analysis, information on the choice of priors and Markov chain Monte Carlo settings
☒	☐	For hierarchical and complex designs, identification of the appropriate level for tests and full reporting of outcomes
☒	☐	Estimates of effect sizes (e.g. Cohen's *d*, Pearson's *r*), indicating how they were calculated

Our web collection on statistics for biologists contains articles on many of the points above.

Software and code

Policy information about availability of computer code

Data collection	SerialEM, version 3.8 was used for data colletion, https://bio3d.colorado.edu/SerialEM/.<br>Computational modeling was performed with NAMD 2.12 and 2.15 (https://www.ks.uiuc.edu/Research/namd/), SILCS 2020.2 (https://silcsbio.com/software/), Gromacs 2018 (https://manual.gromacs.org/documentation/), CGenFF 2.3.0 (https://silcsbio.com/software/), Anton 2 software version 1.27.0 (doi: 10.1109/SC.2014.9), CHARMM-GUI v3.1 server (http://www.charmm-gui.org/).
Data analysis	Eman v1.9 was used to select micrographs manually, https://blake.bcm.edu/emanwiki/EMAN2; RELION 3.0, https://github.com/3dem/relion. Computational modelling and data analysis was performed using VMD 1.9.3 (https://www.ks.uiuc.edu/Research/vmd/) and CHARMM v.42b1 (https://www.charmm.org/archive/index.html) with in house scripts, available at https://github.com/hzhekova/Scripts_for_NatComm.

For manuscripts utilizing custom algorithms or software that are central to the research but not yet described in published literature, software must be made available to editors and reviewers. We strongly encourage code deposition in a community repository (e.g. GitHub). See the Nature Research guidelines for submitting code & software for further information.

Data

Policy information about availability of data

All manuscripts must include a data availability statement. This statement should provide the following information, where applicable:

- Accession codes, unique identifiers, or web links for publicly available datasets
- A list of figures that have associated raw data
- A description of any restrictions on data availability

All the data are available upon reasonable request following publication. The final cryoEM map of NDCBE is submitted to the Electron Microscopy Data Bank (EMDB) under the accession code EMD-24683 that will be released following publication. The final atomic model is submitted to the Protein Data Bank (PDB) under the accession code 7RTM that will be released following publication.

Field-specific reporting

Please select the one below that is the best fit for your research. If you are not sure, read the appropriate sections before making your selection.

- [x] Life sciences
- [ ] Behavioural & social sciences
- [ ] Ecological, evolutionary & environmental sciences

For a reference copy of the document with all sections, see nature.com/documents/nr-reporting-summary-flat.pdf

Life sciences study design

All studies must disclose on these points even when the disclosure is negative.

Sample size	Transport studies: The number of experiments is described under Figure Legends. One-Way Anova followed by post-hoc Dunnett's test was used and the number experiments was performed until the p value was highly significant at p<0.0001. Cryo-EM studies: The number and identity of the particles that went into each refined map were determined via 3D classification, as described under Methods, Image processing.
Data exclusions	Transport studies: No data exclusion was performed. Cryo-EM studies: Micrographs for which motion correction and ctf fitting were applied, were selected manually by discarding apparent bad ones.
Replication	Transport studies: The number of times each construct was studied is described under Figure Legends. Cryo-EM studies: No replication was performed and all analysis algorithms were deterministic.
Randomization	Transport studies: Randomization was not done per se. The function of the wild-type transporter was compared statistically to each mutant transporter in these studies. This is the method typically used in these type of functional studies by all investigators. Cryo-EM studies: During auto-3D refinement, data were randomly split into 2 groups following the "gold standard" protocol (doi:10.1038/nmeth.2115), which generated half1 and half2 maps to enable resolution estimation through cross-validation.
Blinding	Transport studies: No blinding was done as the protocols during the data collection needed to be known to perform the studies properly and address any difficulties during data collection. Cryo-EM studies: No blinding was performed as the exact identity of the sample needed to be known for the proper analysis of each sample.

Reporting for specific materials, systems and methods

We require information from authors about some types of materials, experimental systems and methods used in many studies. Here, indicate whether each material, system or method listed is relevant to your study. If you are not sure if a list item applies to your research, read the appropriate section before selecting a response.

Materials & experimental systems

n/a	Involved in the study	
☐	☒	Antibodies
☐	☒	Eukaryotic cell lines
☒	☐	Palaeontology and archaeology
☒	☐	Animals and other organisms
☒	☐	Human research participants
☒	☐	Clinical data
☒	☐	Dual use research of concern

Methods

n/a	Involved in the study	
☒	☐	ChIP-seq
☒	☐	Flow cytometry
☒	☐	MRI-based neuroimaging

Antibodies

Antibodies used	NDCBE antibody NDCBE-1r was generated and characterized in our previous study (J Comp Neurol. 2012 May 1; 520(7): . doi:10.1002/cne.22806). Secondary Peroxidase AffiniPure Mouse Anti-Rabbit IgG (H+L) was from Jackson Immunoresearch, cat. # 211-035-109. https://www.jacksonimmuno.com/catalog/products/211-035-109.
Validation	The NDCBE antibody is a polyclonal antibody raised in rabbits and was raised against the C-terminal sequence LSINSGNTKEKSPFN that is identical in rat, mouse and human NDCBE. NDCBE-1r recognizes rat, mouse and human NDCBE. It works on immunocytochemistry, Western blotting and pull-down experiments.

Eukaryotic cell lines

Policy information about cell lines

Cell line source(s)	HEK293 human embryonic kidney cell line was purchased from the ATCC: 293 [HEK-293] (ATCC® CRL-1573™).
Authentication	Authentication was performed by ATCC.

Mycoplasma contamination	No mycoplasma contamination purity was stated by the provider.
Commonly misidentified lines<br>(See ICLAC register)	None
